# Identification of chemical scaffolds for targeting ubiquitin-specific protease 11 (USP11) through high-throughput virtual screening

**DOI:** 10.1080/14756366.2025.2518191

**Published:** 2025-06-30

**Authors:** Hobin Lee, Sunghoon Hurh, Soomin Kang, Jihwan Yoon, Jong-Ik Hwang, Derek T. Logan, Hong-Rae Kim

**Affiliations:** ^a^ Laboratory of Discovery Chemistry, Department of Biomedical Sciences, Korea University College of Medicine, Seoul, Republic of Korea; ^b^ GPCR & Signal Transduction Laboratory, Department of Biomedical Sciences, Korea University College of Medicine, Seoul, Republic of Korea; ^c^ Section for Biochemistry and Structural Biology, Centre for Molecular Protein Science, Department of Chemistry, Lund University, Lund, Sweden

**Keywords:** Ubiquitin-specific protease, inhibitor, virtual screening, protein-ligand interactions

## Abstract

USP11 is a promising therapeutic target implicated in Alzheimer’s disease and various cancers; however, no specific inhibitors are currently available, with the only known inhibitor being mitoxantrone, which primarily targets topoisomerase II. To identify novel chemical starting points, we conducted high-throughput virtual screening using a USP11 homology model. Screening over 600,000 compounds yielded five structurally distinct hits with significant inhibitory activity. Biochemical validation highlighted two promising scaffolds: benzoxadiazole derivatives and pyrrolo-phenylamidine analogues, both demonstrating structure-dependent inhibition and tractable SAR profiles. Docking studies further characterised their binding modes, supporting their potential for optimisation. Hydroxyphenyl hydrazone analogues raised PAINS-related concerns, while compounds such as squalamine were deprioritized due to weak binding affinity and structural complexity. Overall, this study provides valuable scaffolds and mechanistic insights that can inform future development of potent, selective USP11 inhibitors.

## Introduction

The ubiquitin-proteasome system (UPS) plays a central role in maintaining protein homeostasis, and its dysregulation is linked to numerous human diseases^1–6^. Ubiquitination, a reversible process, involves the attachment of ubiquitin to target proteins by the E3 ubiquitin ligase complex^7^, while deubiquitinating enzymes (DUBs) mediate the removal of ubiquitin from their client proteins^8^. Polyubiquitination is primarily associated with marking proteins for degradation via the proteasome, whereas mono-ubiquitination regulates key signalling pathways^9^. By reversing ubiquitination, DUBs tightly regulate these processes, maintaining cellular and physiological balance^10^^,^^11^.

The ubiquitin-specific protease (USP) family, which includes over 50 identified members, is the largest group of DUBs and holds great therapeutic promise^12^^,^^13^. USP7, for instance, has been extensively studied for its role in the p53 tumour suppressor pathway, leading to the development of several potent and selective inhibitors^14^. While many other members of this family are also being investigated for their therapeutic potential, the lack of chemical tools to modulate these targets has posed significant challenges for further exploration^15^^,^^16^.

USP11 is one such DUB with considerable therapeutic promise but remains underexplored due to the absence of specific chemical inhibitors. In a recent study, Yan et al. identified X-linked USP11 as a key driver of tauopathy vulnerability in women, deubiquitinating the tau protein and promoting tau acetylation and aggregation^17^. Furthermore, USP11 has been implicated in multiple cancers^18^, including colorectal^19^ and breast cancer^20^^,^^21^, as well as graft-versus-host disease^22^. Despite its significance, the only known small molecule inhibitor of USP11 to date is the FDA-approved drug mitoxantrone, which primarily targets topoisomerase II^23^. Mitoxantrone also inhibits USP15^24^, a close homologue of USP11, complicating the study of USP11-specific functions. Developing a potent and selective USP11 inhibitor could not only advance the understanding of USP11’s biological roles but could also provide a promising foundation for therapeutic applications.

Given the current lack of specific inhibitors for USP11, we conducted a high-throughput virtual screening to identify potential starting points for USP11 inhibitor discovery. This screening effort identified several structurally unique chemical scaffolds with the potential to serve as foundations for medicinal chemistry campaigns. To better understand the binding mechanisms, we analysed *in silico* the protein-ligand interactions of these molecules with USP11. Collectively, these findings provide valuable insights for the medicinal chemistry community, enabling structure-based design for USP11 and contributing to the development of potent and selective USP11 inhibitors.

## Materials and methods

### Chemicals

All compounds and their structural analogues evaluated in this study were sourced exclusively from the Korea Chemical Bank (KCB), which comprises compounds from commercial vendors, bioactive molecules, and natural products. Mitoxantrone and squalamine were purchased from a commercial vendor (HY-13502, HY-16468, MedChemExpress, USA).

### Cloning, expression, and purification of GST-USP11

The plasmid containing the human USP11 gene was purchased from Sino Biological Inc. (Beijing, China). PCR products of the gene were digested and inserted into EcoRI/XhoI sites in the pGEX4T-1 prokaryote expression vector (Cytiva Life Sciences, Marlborough, MA, USA). After ligation, the construct was transformed into *E. coli* DH5α. The DH5α strain, originally obtained from Thermo Fisher Scientific (Waltham, MA, USA), was made competent in our lab for plasmid transformation. The isolated plasmids containing the USP11 gene were sequenced with several internal primers (Cosmo Genetech Co., Ltd., Seoul, Korea). The plasmids containing the intact USP11 gene were introduced into *E. coli* BL21-CodonPlus (DE3) for protein expression (Invitrogen, Carlsbad, CA, USA). The bacteria were cultured overnight in LB broth containing 100 μg/ml ampicillin and used to inoculate 2xYT expression broth at a ratio of 1:10 relative to cultured volume. The broth was cultured at 37 °C with shaking at 200 rpm until the OD_600_ was 0.5. After cooling down on ice, 0.1 mM isopropyl-β-D-thiogalactopyranoside was added to the broth, which was cultured at 25 °C at 200 rpm for 5 h. The bacteria were collected by centrifugation at 5,000 rpm for 10 min and lysed with a buffer containing 50 mM Tris-HCl, pH 7.5, 150 mM NaCl, 1% Tx-100, and protease inhibitor cocktail. After removing undissolved parts by centrifugation at 15,000 rpm at 4 °C for 20 min, the supernatants were incubated with appropriate amount of GSH sepharose 4B from Cytiva (Marlborough, MA, USA). After 1 h nutation at 4 °C, the reactants were transferred to Econo-column^®^ Chromatography Column (Bio-rad, Hercules, CA, USA). Non-binding proteins were washed out by the lysis buffer and PBS. The GST-USP11 proteins were eluted by 10 mM reduced GSH, dialysed with PBS overnight, and concentrated using Amicon Ultra centrifugal filters (Merk Millipore, Burlington, MA, USA).

### Fluorescence-based deubiquitinating enzyme activity assay

The deubiquitinating enzyme (DUB) activity of USP11 was monitored using Ubiquitin AMC (Ub-AMC) (#U-550–050, R&D Systems, Minneapolis, MN, USA) as a fluorogenic substrate. Purified GST-USP11 (100 nM) was pre-incubated with 25 μM of each tested compound or DMSO (vehicle control, 0.5% v/v) in DUB assay buffer (50 mM HEPES, 0.5 mM EDTA, 1 mM DTT, and 0.1 mg/ml BSA) at room temperature for 30 min. Ub-AMC (100 nM) was then added and DUB activity was quantified for 1.5 h at 10 min intervals by measuring fluorescence (excitation 345 nm; emission 445 nm) upon the cleavage of Ub-AMC over time at 37 °C in a FlexStation 3 microplate reader (Molecular Devices, San Jose, CA, USA). USP11 DUB activity of vehicle control or each compound group was quantified by calculating the area under curve (AUC) using Prism 9 software (GraphPad, Boston, MA, USA).

### Biolayer interferometry (BLI) assay

The binding affinities of tested compounds for USP11 were determined using BRAND^®^ microplates (#BR781608, Merck, Darmstadt, Germany) and an Octet^®^ R8 system (Sartorius AG, Göttingen, Germany). Octet GST Biosensors (#18–5096, Sartorius AG, Göttingen, Germany) captured with purified GST-USP11 (50 μg/ml) or without proteins were used as ligand sensors or reference sensors, respectively. For binding kinetic measurements, the association and dissociation of tested compounds (3.125–100 μM) in assay buffer (PBS, 0.02% Tween 20, 2% DMSO) were monitored at 25 °C with 1,000 rpm stirring for 90 s. For accurate baseline, wells containing assay buffer only were used as reference wells. All data were analysed by Octet BLI analysis software version 12.2 (Sartorius AG, Göttingen, Germany). The binding graphs were obtained using a double reference subtraction protocol and equilibrium dissociation constant (*K*_D_) values were calculated from the ratio of the dissociation rate constant (*K*_dis_) to the association rate constant (*K*_on_).

### In silico experiments

All *in silico* experiments were performed in a Windows OS environment running on a HP EliteDesk 800 G9 TWR Workstation. Pre-processing for molecular docking (Protein Preparation Wizard, LigPrep, Receptor Grid Generation), virtual screening (Glide), visualisation (Maestro), and analysis were conducted with Schrödinger’s Small Molecule Drug Discovery Suite (version 2022–4). For decoy generation, the SMILES string of mitoxantrone was submitted for DUD-E generation (https://dude.docking.org/), resulting in 50 unique decoys. For homology model generation, the human USP11 sequence (UniProt ID: P51784) was retrieved in FASTA format from the UniProt database and imported into Schrödinger’s Homology Modelling platform as the target sequence. USP15 template structures (PDB IDs: 6CPM, 6CRN, 6GH9, 6ML1) were obtained from the Protein Data Bank (PDB). Sequence alignment was performed, ensuring proper alignment of the catalytic cysteine residues (Cys318 for USP11 and Cys298 for USP15). Ligands and cofactors were excluded during the generation of the homology models. Generated homology models were loaded into Maestro, and the proteins were prepared for docking using the Protein Preparation Workflow^25^ module. Missing side chains and loops were filled in with Prime^26^, and the PROPKA^27^ module was used for optimisation of the protonation states. Minimisation was performed with the OPLS4 force field and the water molecules less than 5 Å from the ligand were removed. All ligands were prepared using Schrödinger’s LigPrep module with the OPLS4 force field. All possible states at a target pH of 7.0 ± 2.0 were generated using Epik^28^, and only the specified chiralities, if known, were retained. For compounds without chirality information, all possible isomers were generated. A receptor grid 20 Å on each side was generated having Cys318 as the centroid. Ligands were docked using Glide, with varying precision. In all cases, the ligand docking was conducted using van der Waals radii scaling factor of 0.80 with partial charge cut-off at 0.15. Ligand sampling was left flexible, and Epik state penalties were applied to docking scores. For the Virtual Screening Workflow (VSW), the ligands were pre-prepared using the LigPrep function and therefore the ligand preparation step was skipped. No filtering was applied in any of the VSW experiments. Glide HTVS docking was unselected from the workflow and only the top 1% of the XP docking was retained.

## Results

### Structure-based virtual screening with the USP11 homology model

At the time this project was initiated, no experimental structure of USP11 had been solved; therefore, the screening was conducted using predicted structural models. We generated four homology models based on USP15, which shares the closest sequence similarity with USP11 (51.2% sequence identity for the catalytic domain), and also evaluated the AlphaFold model of USP11 (AF-P51784-F1-model_v4). To benchmark these models, we assessed their ability to identify mitoxantrone, a known USP11 inhibitor, as a positive control among DUD-E-generated decoys^29^. Ideally, having multiple known binders and calculating the enrichment factor (EF) to assess each model’s ability to discriminate binders from decoys would have strengthened benchmarking confidence^30^. However, with only one known inhibitor available, our primary focus was to identify a model that ranked mitoxantrone as the highest-scoring compound within the screening set. Mitoxantrone served strictly as a tool to assess the reliability of each homology model, rather than to guide hit selection. The primary goal of the screening was to identify novel and structurally diverse scaffolds with potential for USP11 inhibition. Accordingly, the virtual screening campaign prioritised chemical diversity and scaffold novelty to support future medicinal chemistry optimisation.

The virtual screening utilised Schrödinger’s Virtual Screening Workflow (VSW) protocol, which begins with high-throughput virtual screening (HTVS) precision, prioritising speed over accuracy. The top 10% of compounds from HTVS are subsequently screened with standard precision (SP), and the top 10% of SP-ranked compounds are further refined using extra precision (XP), a more thorough but time-intensive method, to yield the final candidates. Since the final candidates are derived from the XP screen, we focused on models that could prioritise mitoxantrone in this stage of the workflow.

We began by performing an XP-precision screening of the decoy set to ensure that at least one of the structures can capture mitoxantrone as the highest-ranking compound. As shown in Table 1, the homology model based on 6CRN identified mitoxantrone as the top compound in the XP screening. However, in a pilot VSW screening, none of the structures retained mitoxantrone. This was due to the 6CRN-based model’s inability to rank mitoxantrone within the top 10% during HTVS precision screening, resulting in its exclusion early in the virtual screening process. Despite this limitation, mitoxantrone ranked within the top 10% of SP compounds, allowing it to proceed to the XP screening. To address this issue, we modified the default VSW protocol to skip the HTVS precision step and use SP screening as the initial stage. To compensate for the removal of this filtration stage, we further refined the XP screening results by selecting only the top 1% of compounds, instead of the top 10%, as final candidates.

**Table 1.** Evaluation of homology models for their ability to identify the positive control mitoxantrone among decoys.

Model Based on	X-Ray Diffraction Resolution (Å)	USP Complexed with	HTVS Precision		Standard Precision (SP)		Extra Precision (XP)	
			MTX Rank	Docking Score	MTX Rank	Docking Score	MTX Rank	Docking Score
6CPM	2.011	Ubiquitin variant	3	−3.848	1	−5.997	2	−6.193
6CRN	2.5	Ubiquitin variant	>10	N/A	5	−6.456	1	−11.09
6GH9	2.09	Mitoxantrone	2	−4.219	14	−4.631	10	−5.531
6ML1	1.9	Ubiquitin variant	>10	N/A	11	−4.803	2	−6.588
Alpha Fold*	Predicted	apo structure	1	−5.689	3	−5.619	2	−6.557

Decoys were generated using DUD-E, and rankings were determined based on docking scores. *AlphaFold (AF-P51784-F1-model_v4) model was used.

For the screening, we utilised the Korea Chemical Bank (KCB) library, a diverse collection containing 633,030 compounds (as of 2023) from commercial vendors, bioactive molecules, and natural products, available in small quantities suitable for experimental validation. The initial top 600 compounds identified from virtual screening were clustered primarily based on structural similarity to ensure chemical diversity for subsequent evaluation. Each cluster was visually inspected, and representative compounds were selected for biochemical validation. While docking scores were the primary criterion for selection, structural clustering helped prioritise distinct chemical scaffolds. Although Lipinski’s Rule of Five is widely considered essential for potential drug screening, we intentionally avoided strictly applying this rule at this early stage to prevent prematurely excluding potentially promising compounds. This rationale aligns with recent evidence demonstrating that many FDA-approved drugs do not strictly adhere to these guidelines^31–33^. Instead, to balance practicality and inclusivity, we excluded only compounds clearly lacking lead-like characteristics (e.g. molecular weight below 200), resulting in 77 final clusters for biochemical validation.

### Biochemical evaluation of the compounds derived from virtual screening

**Figure 1.**
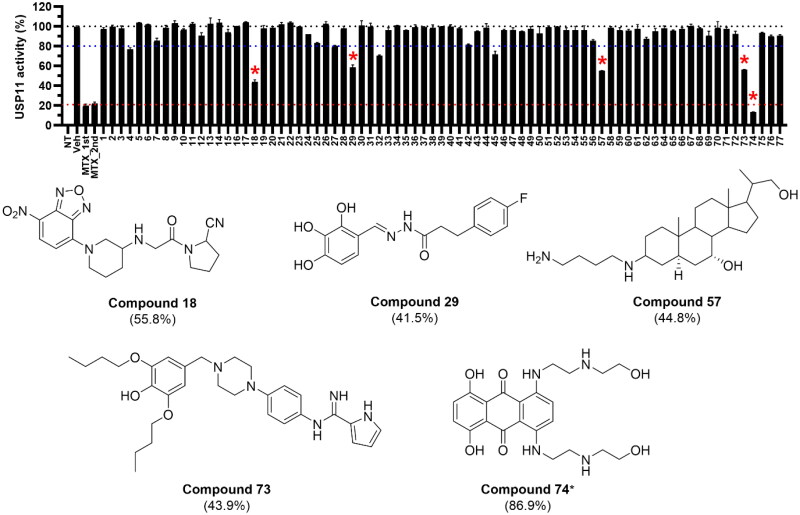
Inhibition of USP11 by virtual screening-derived compounds measured using a fluorogenic substrate assay. A total of 77 compounds were evaluated at a fixed concentration of 25 µM, with percent inhibition measured at the 90-min endpoint. Percent inhibition is indicated in brackets. *Compound **74** corresponds to mitoxantrone.

We first validated that our expressed and purified GST-fused USP11 protein was catalytically active and that its activity could be measured in a dose-dependent manner. Using a ­fluorogenic substrate assay with ubiquitin 7-amido-4-methylcoumarin (Ub-AMC), we tested varying concentrations of both the protein and the substrate. The results confirmed that our assay setup reliably measures enzymatic activity (Figure S1). Subsequently, we tested the 77 compounds derived from the virtual screening for their inhibitory potential against USP11 at a fixed concentration of 25 μM. Hits were defined as compounds that inhibited enzyme activity by more than 40%, as determined by fluorescence intensity at the 90-min endpoint. Five unique structures were identified as hits, including mitoxantrone, which was part of the KCB library (Figure 1). Notably, three of these compounds (**57**, **73**, and **74**) contained alkyl chains, suggesting that this moiety may play an important role in USP11 inhibition.

### Structure-activity relationship studies of mitoxantrone and pyrogallol hydrazone analogues

The SAR-by-catalogue approach is an efficient strategy for collecting initial structure-activity relationship (SAR) data to guide the design of compounds for synthesis. In this study, compounds were grouped into clusters based on structural similarity, with a single representative from each cluster tested for activity in initial assays. After identifying active representatives, structural analogues within the same KCB library clusters were subsequently tested to gain deeper insights into their structure-activity relationships. This comprehensive approach allowed us to evaluate whether any analogues displayed improved potency over the initially tested compounds, providing deeper insights into the structural features that merit further investigation.

**Figure 2.**
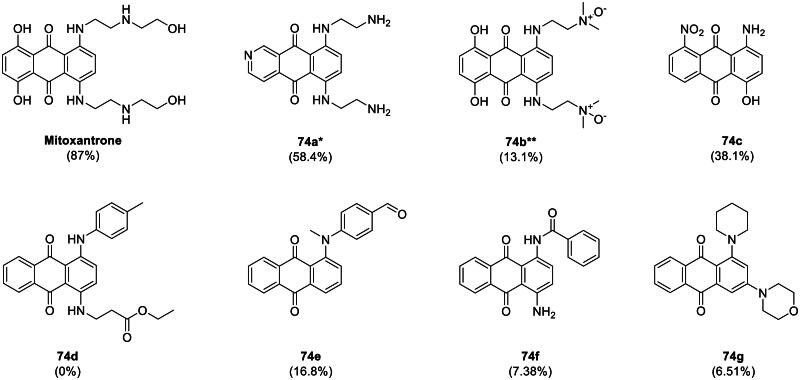
SAR-by-catalogue study of mitoxantrone analogues. A total of seven analogues were evaluated at a fixed concentration of 25 µM, with percent inhibition measured at the 90-min endpoint. Percent inhibition is indicated in brackets. * Indicates the sample was provided in the 2 maleic acid salt form; ** indicates the 2 hydrochloric acid salt form.

We began by investigating mitoxantrone (compound **74**) and its structural analogues to evaluate their potential as USP11 inhibitors. Seven analogues were evaluated for their inhibitory activity against USP11 (Figure 2); unfortunately, none of the tested compounds demonstrated greater potency than mitoxantrone.

A brief structure-activity relationship (SAR) analysis suggested that linear side chains are critical for activity, as compounds lacking them were inactive. Specifically, replacing the linear side chain with cyclic structures, whether aromatic or aliphatic, led to a loss of activity, indicating that the straight, non-cyclic nature of mitoxantrone’s side chains plays a crucial role. This hypothesis is further supported by the observation that compound **74b**, which contains a bulkier dimethylamine moiety at the end of the side chain, exhibited a significant reduction in activity. Similarly, compound **74a**, with a shorter side chain, showed reduced activity compared to mitoxantrone. However, since **74a** also replaces the dihydroxyphenyl group of mitoxantrone with a pyridine moiety, the reduced activity cannot be solely attributed to the shorter side chain.

While the tricyclic core with a linear side chain remains a promising template for USP11 inhibitor discovery, it is important to acknowledge that mitoxantrone’s primary target is topoisomerase II. Consequently, future studies should evaluate whether compounds derived from this scaffold also inhibit topoisomerase II as a potential off-target effect.

As we reviewed analogues related to compound **29**, we observed that all structures shared a characteristic hydroxyphenyl hydrazone moiety, a well-known Pan-Assay Interference Compound (PAINS) structural alert^34^. During the design of the virtual screening, we considered applying the PAINS filter to exclude compounds with features commonly associated with promiscuous activity, but treated it as a guideline rather than a strict rule, recognising that rigid application could unnecessarily exclude potentially promising compounds^35^. Consequently, instead of automatically excluding compounds flagged by the PAINS filter, we conducted manual inspections to determine whether the flagged features might misleadingly influence activity. For the pyrogallol hydrazone series, SAR-by-catalogue screening revealed nearly identical inhibitory activity (approximately 50%) across all compounds, regardless of structural variation (Figure S2). This consistent activity suggested that the observed inhibition may not result from specific interactions with USP11 but rather from a non-specific mechanism associated with the shared hydroxyphenyl hydrazone moiety.

While the lack of structural variability in activity raises concerns about potential PAINS-related promiscuity, the evidence is insufficient to definitively attribute this behaviour solely to such characteristics, as structural elements outside the hydroxyphenyl hydrazone may play only a minimal role in binding. Although experimental validation could provide further clarity, we decided not to pursue this scaffold further and instead focused on scaffolds with clearer structure-activity relationships and greater potential for developing selective and potent USP11 inhibitors.

### Marine natural product squalamine inhibits USP11 but with weak binding affinity

In our search for structural analogues of compound **57** within the KCB library, the steroid structure of the parent compound yielded numerous “hits” through an SAR-by-catalog approach. Ten structurally similar compounds were identified, with most, except for compounds **57a** and **57i**, demonstrating complete inhibition of USP11 at 25 μM (Figure S3). However, no clear structure-activity relationship (SAR) trends were observed, raising concerns about the reliability of the observed activity and the possibility of false positives.

One potential confounding factor was that many of the analogues were supplied as 3- or 4-HCl salt forms, raising the concern that shifts in pH might alter the protonation state of the catalytic cysteine, thereby affecting enzyme activity. However, pH measurements confirmed that the assay buffer system remained stable, effectively ruling out pH-induced artefacts. To further evaluate the validity of the observed inhibition, biophysical experiments with compound **57j** confirmed direct target engagement, with a *K*_D_ value of 58.5 μM as measured by biolayer interferometry (Figure S4).

Despite this confirmation, efforts to rationalise activity based on structural features remained inconclusive. For example, we initially hypothesised that the hydroxyl group at C7 was detrimental to activity, given the reduced potency of compounds **57a** and **57b**; however, compound **57d** retained high potency despite the presence of the C7 hydroxyl group. This led us to consider the possible contribution of the extended aminoalkyl chain in **57d**, yet compound **57e**, which lacks the C7 hydroxyl group, also remained active. These findings suggest that both the aminoalkyl chain and the C7 hydroxyl group may influence potency in distinct or potentially synergistic ways, underscoring the complexity of the SAR.

Ultimately, the major limitation in deciphering these trends was the absence of stereochemical information. The stereochemistry of the steroidal core, particularly the *cis*- or *trans*-fusion of the rings, profoundly influences the three-dimensional conformation and, by extension, biological activity. Unfortunately, the lack of stereochemical data in the KCB library severely hindered our ability to establish definitive SAR conclusions.

**Figure 3.**
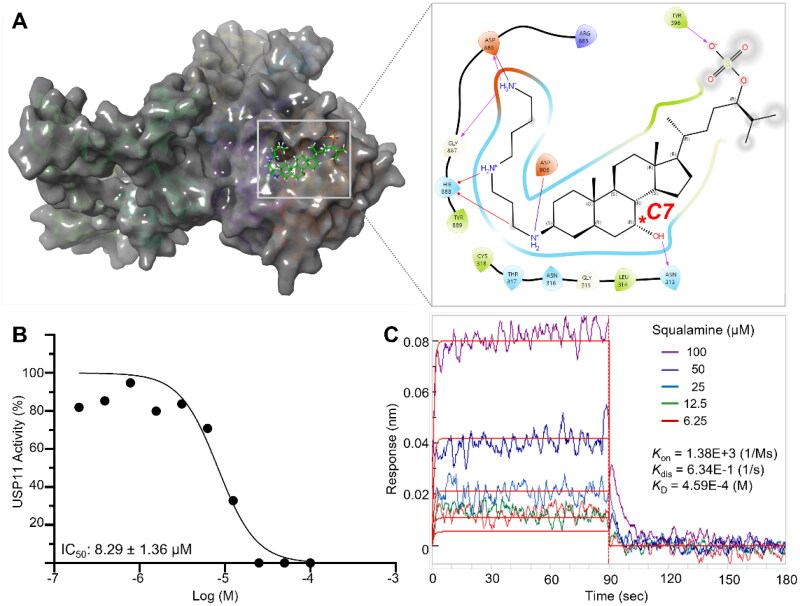
Squalamine inhibits USP11. (a) Protein-ligand interaction between USP11 and squalamine determined by molecular docking analysis. (b) Inhibitory activity of squalamine against recombinant USP11. IC_50_ values were calculated based on measurements at 0.2, 0.39, 0.78, 1.56, 3.13, 6.25, 12.5, 25, 50 and 100 μM. (c) Binding affinity was measured with biolayer interferometry with an anti-GST biosensor at five different concentrations.

Given these limitations, we shifted our focus to a structural analogue with well-defined stereocenters. Squalamine, a marine natural product, was identified as a close analogue of compound **57** featuring a spermidine group at C3 and clearly defined stereochemistry, making it a suitable model for further investigation (Figure 3(a)). We confirmed that squalamine completely inhibits USP11 at 25 μM and, when compared with the positive control mitoxantrone, found squalamine to be a more potent inhibitor at the recombinant protein level (IC_50_ = 8.29 μM for squalamine vs. 13.3 μM for mitoxantrone, Figure 3(b), Figure S1C).

Building on these findings, we further characterised the binding properties of squalamine using biophysical experiments (Figure 3(c)). The analysis revealed a dose-dependent interaction, although the binding affinity was relatively weak (*K*_D_ = 459 µM). This was largely attributed to the compound’s slow binding kinetics (*K*_on_ = 1.38 × 10³ M^−1^s^−1^) and rapid dissociation (*K*_off_ = 6.34 s^−1^). While such characteristics could be advantageous in scenarios requiring fast and reversible inhibition, the weak binding affinity, combined with the complexity of the steroid structure, led us to conclude that this series of compounds are not ideal candidates for further structure-activity relationship (SAR) exploration. Consequently, we decided not to pursue its development further.

### Structure-activity relationship and docking insights of the benzoxadiazole analogues

**Figure 4.**
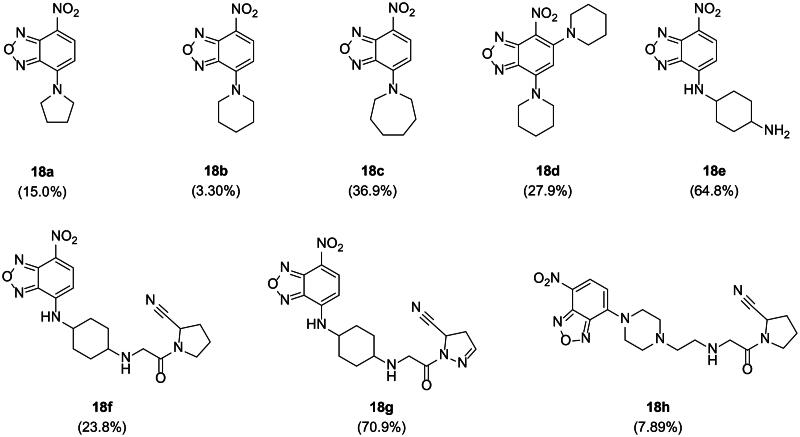
SAR-by-catalogue study of the benzoxadiazole analogues. Percent inhibition of USP11 at a compound concentration of 25 µM, determined using a fluorogenic substrate assay, is shown in brackets.

The structure-activity relationship analysis of eight benzoxadiazole (compound **18**) analogues revealed structure-dependent variations in activity (Figure 4), suggesting that this series may serve as a promising template for developing USP11 inhibitors. A comparison of compounds **18a**, **18b**, and **18c** demonstrated a clear preference for larger cyclic structures, implying that the binding site can accommodate bulky cyclic moieties. Notably, the introduction of a nitrogen spacer between the benzoxadiazole core and the cyclohexyl ring (**18e**) significantly enhanced activity, highlighting opportunities for structural optimisation at this site.

Interestingly, compounds **18f** and **18 g**, featuring cyanopyrrolidine and cyanopyrazole moieties, respectively, exhibited markedly different activities despite subtle structural differences. To better understand this observation, we compared the docked poses of the two ligands. Given that both compounds possess two chiral centres in the cyclohexyl moiety and can exist in different protonation states, we calculated the MM-GBSA (Molecular mechanics with generalised Born and surface area solvation) scores for all possible isomeric states and selected the best-scoring poses for analysis. Based on the MM-GBSA binding free energy (Δ*G*_bind_), compound **18 g** (−51.43 kcal/mol) formed a more stable complex than **18f** (−49.99 kcal/mol), with the *cis*-configuration of the chiral centres being favoured in both cases.

**Figure 5.**
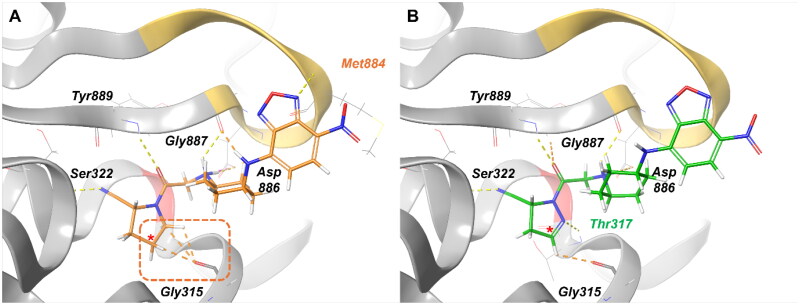
Comparison of docking poses between (a) **18f and (b) 18 g.** Yellow dashed lines represent hydrogen bonding interactions, while the orange dashed box and lines highlight steric clashes observed in **18f**. Residues interacting uniquely with each ligand are marked in orange (Met884 for **18f**) and green (Thr317 for **18 g**). A red asterisk (*) denotes the *sp³* versus *sp^2^* carbon uniquely present in each ligand. The yellow loop indicates the blocking loop 2 (BL2, residues 881–887) and red residue indicates Cys318.

The docking poses of **18f** and **18 g** revealed overlapping features but with key differences (Figure 5, S5). In **18f**, the *sp*^3^ hydrogens created steric clashes with Gly315, potentially hindering optimal binding. This issue was absent in **18 g**, where the *sp^2^* hydrogen at the equivalent position avoided steric interference. Additionally, the pyrazole group in **18 g** formed a hydrogen bond with Thr317, further stabilising its complex.

The reduced activity of **18h**, which features a piperazine directly attached to the benzoxadiazole core, suggests that multiple factors, such as the distance between the benzoxadiazole and the cyanopyrrolidine, as well as the conformation of the central six-membered ring, play significant roles in binding efficacy. Docking analysis indicated that the benzoxadiazole moiety resides in a solvent-exposed region of the binding site, suggesting it could be further modified to enhance interactions. Supporting this, compound **18d**, which incorporates a piperidine moiety adjacent to the nitro group, displayed improved activity compared to **18a**.

### Pyrrolo-phenylamidines as a potential scaffold for USP11 inhibitors

**Figure 6.**
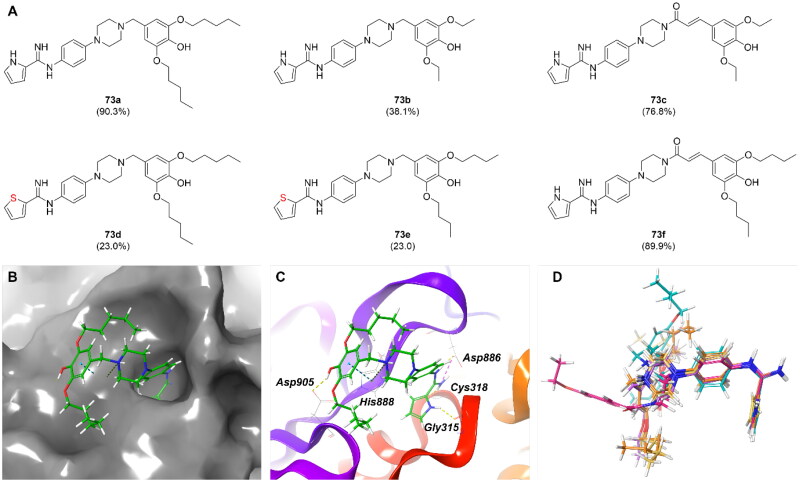
SAR-by-catalogue study of pyrrolo-phenylamidine analogues. (a) Structures and percent inhibition of USP11 at a compound concentration of 25 µM, determined using a fluorogenic substrate assay, is shown in brackets. Molecular docking studies of **73a** complexed with USP11 presented on (b) surface map and (c) protein-ligand interaction. (d) Superimposition of binding poses of compounds **73a** to **73e**.

The pyrrolo-phenylamidine series of compounds (**73**) were previously reported as antioxidants^36^; however, our study revealed their potential as templates for USP11 inhibitors (Figure 6(a)). A comparison of compounds **73**, **73a**, and **73b** demonstrated that the length of the alkyl chain significantly influences activity, with longer chains correlating with increased potency. This trend was further supported by **73c** and **73f**, where **73f**, featuring a two-carbon elongation relative to **73c**, exhibited greater activity.

The pyrrole moiety appears to play a critical role in binding, as replacing it with a thiophene led to a significant loss of activity, as observed in **73d** and **73e**. Interestingly, the linker connecting the piperazine to the 3,5-dialkoxy-4-hydroxyphenyl moiety, whether a methyl group or an unsaturated ketone, did not affect potency, as evidenced by the similar activities of **73a** and **73f** (IC_50_ = 17.49 uM and 19.38 uM, respectively, Figure S6). This observation suggests that the 3,5-dialkoxy-4-hydroxyphenyl moiety may not form specific interactions but instead occupies a broader space where hydrophobicity and rotatable bonds drive binding.

Molecular docking studies supported the experimental findings. With the exception of **73f**, all compounds exhibited amidine interactions with Asp886 and the backbone of Cys318. Pyrrole-containing compounds formed additional hydrogen bonds with Gly315, which were absent in thiophene-containing analogues. Notably, the 3,5-dialkoxy-4-hydroxyphenyl moiety was positioned in a solvent-exposed region, aligning with the hypothesis that it contributes less to specific binding (Figure 6(b, c)). Superimposition of the binding poses of **73a** to **73e** showed a highly overlapping pyrrolo-phenylamidine core, but with significant deviations observed in the positioning of the 3,5-dialkoxy-4-hydroxyphenyl group (Figure 6(d), Figure S7).

These findings suggest that pyrrolo-phenylamidines represent a promising scaffold for USP11 inhibitors. The structural insights gained from this study highlight opportunities for structure-based design of novel molecules with enhanced binding properties and an optimised fit within the USP11 binding pocket.

### Discussion

In this study, we conducted a high-throughput virtual screening against USP11 and identified five structurally unique compounds with greater than 40% inhibition at 25 μM. Structure-activity relationship studies of their analogues provided valuable insights into their observed inhibitory activities, highlighting potential scaffolds for further exploration.

While the use of PAINS as a strict filter in virtual screening remains open to debate, our analysis of compound **29** reinforced the importance of understanding and validating PAINS-associated structures. Careful consideration of these flagged compounds can provide invaluable information, ultimately saving resources by avoiding the pursuit of low-quality scaffolds.

The series of compounds **57** demonstrated that relying solely on single metrics, such as percent inhibition or IC_50_, is insufficient for fully evaluating inhibitor properties. As seen with squalamine, an inhibitor can exhibit a single-digit micromolar IC_50_ value despite having weak binding affinity. This underscores the importance of incorporating additional metrics, such as binding kinetics, to achieve a more comprehensive characterisation of inhibitor potential.

Two additional scaffolds, compounds **18** and **73**, exhibited structure-dependent activity variations, highlighting their potential for further investigation. On a particular note, the nitrile-bearing compound **18** raises the possibility of covalent inhibition, as seen in the cyanopyrrolidine-containing UCHL1 inhibitor IMP-1710, where the nitrile group functions as an electrophilic warhead (Figure S8)^37^. Experimental validation, such as mass spectrometry, would confirm whether compound **18** acts covalently and could open up opportunities for designing covalent inhibitors.

The pyrrolo-phenylamidine scaffold showed promising potential despite its loose fit in the binding pocket, as suggested by both *in silico* and experimental findings. Designing molecules to test the current binding pose hypothesis, such as exploring cyclic structures instead of alkyl chains, would further support the SAR analysis and docking studies conducted in this work.

Despite demonstrating promising inhibitory activity, we decided against advancing compounds **18** and **73** directly into cellular or other *in vitro* assays at this stage due to their moderate potency and potential off-target effects at micromolar concentrations. Our goal of this work was first to identify promising chemical scaffolds through biochemical assays and *in silico* analyses that could be subsequently optimised for greater potency and selectivity towards USP11. This approach aims to avoid ambiguous biological outcomes, such as those observed with mitoxantrone, which, despite USP11 inhibition, primarily targets topoisomerase II. Future studies will focus on developing these scaffolds into potent and selective inhibitors suitable for comprehensive evaluation in cellular systems and advanced *in vitro* assays.

Overall, instead of focusing on a single scaffold, a comprehensive understanding of the binding modes of each scaffold, combined with integrating all available information, could facilitate the design of more effective inhibitors. For example, X26, a previously reported USP7 inhibitor, was found to inhibit USP11 as an off-target^38^. Structural similarities between X26 and the scaffolds identified in this study (Figure S9) may offer valuable insights into binding modes and could aid in the design of selective and potent USP11 inhibitors.

During the course of this project, the crystal structure of USP11 in its catalytically competent form (PDB: 8OYP) was solved^39^, offering critical insights into the active site. Compared to the homology model based on 6CRN that was most successful in docking mitoxantrone in this study, 8OYP revealed small but significant differences in the blocking loop 2 (BL2) region (residues 881–887), a key component of the active site. The BL2 flap in 8OYP is oriented towards the substrate mimic, reflecting a catalytically competent conformation, whereas in the 6CRN homology model it is oriented in the opposite direction, towards mitoxantrone, which could result in a steric clash with one of the extended arms as observed in the crystal structure of the complex with USP15 (6GH9). The same is true of the homology model based on 6CPM, which ranked mitoxantrone in second place even in the HTVS step. The conformations in 6CRN and 6GH9 most likely represent unbound or alternate states. Such variability underscores the challenges of relying solely on homology models for virtual screening.

The reason why the homology model based on 6CRN performed best in identifying mitoxantrone remains unclear. However, it should be noted that a new crystal structure of mitoxantrone in complex with USP15 was published in 2023 (7R2G)^40^. In this model, the arms of mitoxantrone are oriented in the opposite direction, away from the BL2 flap, and no steric clash will occur. Thus there seems to be flexibility in the binding of mitoxantrone. Nevertheless, to bridge the gap between computational predictions and experimental observations, co-crystal structures of USP11 with identified hits would be invaluable. These structures could validate docking predictions, provide insights into binding interactions, and refine virtual screening workflows. Maurer et al. demonstrated that their USP11 construct retains catalytic activity^39^, making it suitable for co-crystallization efforts. While crystallising the protein without a substrate mimic may be challenging, such studies represent a critical step towards developing potent and selective USP11 inhibitors. We are currently working on obtaining co-crystal structures of USP11 with promising inhibitors identified in this study, and these will be pursued in future work.

### Conclusion

In summary, our study identified structurally unique chemical scaffolds with significant USP11 inhibitory potential, emphasising benzoxadiazole derivatives and pyrrolo-phenylamidines as promising templates for further medicinal chemistry optimisation. These findings provide valuable starting points for the future development of potent, selective, and therapeutically relevant USP11 inhibitors.

## Supplementary Material

Supplemental Material

## Data Availability

All data relevant to the findings of this study are included in the article and its supplementary materials.
