# Supplementary material for: Identification of chemical scaffolds for targeting ubiquitin-specific protease 11 (USP11) through high-throughput virtual screening
Source: J Enzyme Inhib Med Chem. 2025 Jun 30;40(1):2518191. doi: 10.1080/14756366.2025.2518191 (PMC12210405; doi:10.1080/14756366.2025.2518191)
Supplement: Supplemental Material [file IENZ_A_2518191_SM7565.pdf]

Supplementary Information

Identification of Chemical Scaffolds for Targeting Ubiquitin-Specific Protease 11 (USP11) through High-throughput Virtual Screening

Hobin Lee[1], Sunghoon Hurh[2], Soomin Kang[1], Jihwan Yoon[1], Jong-Ik Hwang[2]*, Derek T. Logan[3]*, and Hong-Rae Kim[1]*

[1]Laboratory of Discovery Chemistry, Department of Biomedical Sciences, Korea University College of Medicine, Seoul, Republic of Korea

[2]GPCR & Signal Transduction Laboratory, Department of Biomedical Sciences, Korea University College of Medicine, Seoul, Republic of Korea

[3]Section for Biochemistry and Structural Biology, Centre for Molecular Protein Science, Department of Chemistry, Lund University, Lund, Sweden

*Correspondence to: Jong-Ik Hwang, hjibio@korea.ac.kr; Derek T. Logan, derek.logan@biochemistry.lu.se; Hong-Rae Kim, hrkim07@korea.ac.kr

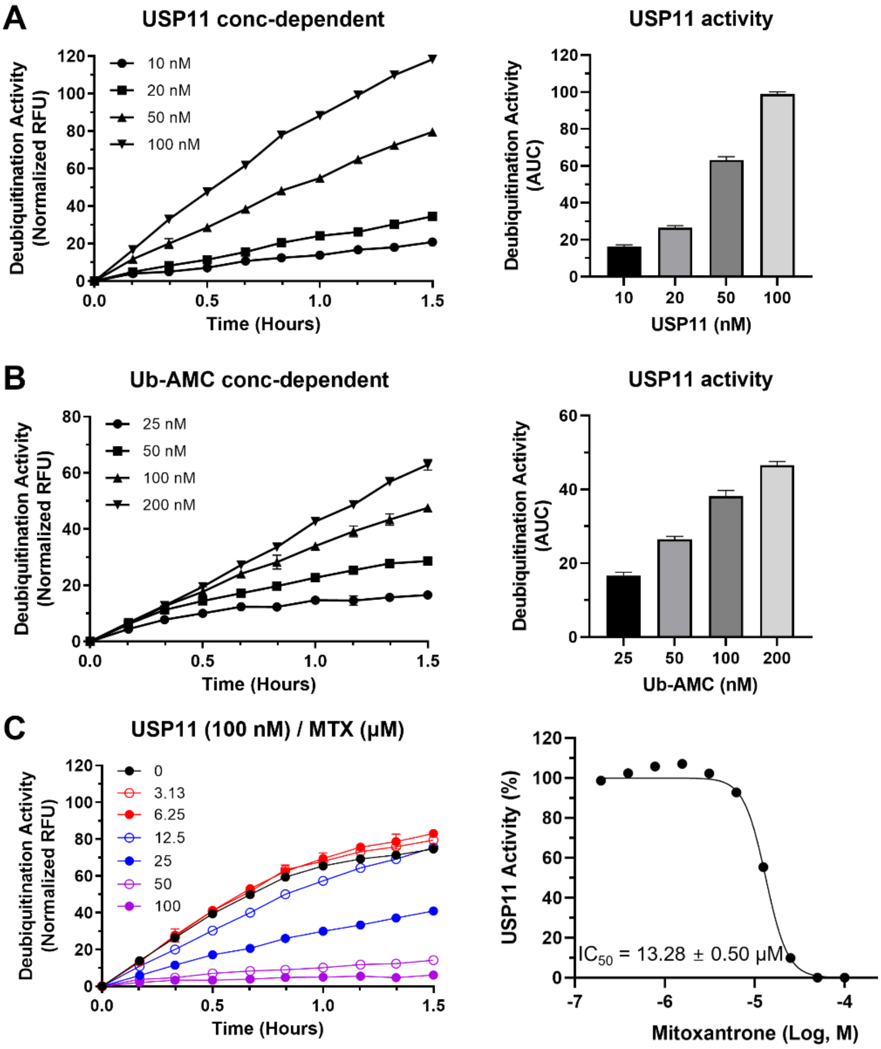

Figure S1. Validation of catalytic activity for expressed and purified GST-fused USP11. A fluorogenic substrate assay using Ubiquitin 7-amido-4-methylcoumarin (Ub-AMC) demonstrated dose-dependent activity with varying concentrations of the **(A)** protein and **(B)** substrate. **(C)** Dose-dependent inhibition by the positive control mitoxantrone.

Information Classification: General

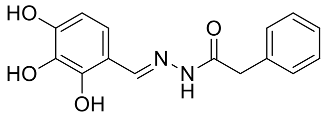

29a
(54.0%)

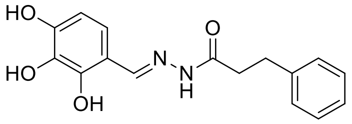

29b
(54.1%)

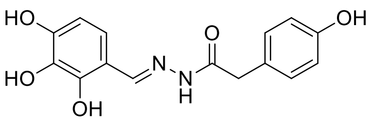

29c
(58.8%)

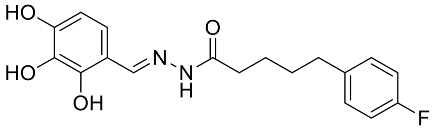

29d
(54.5%)

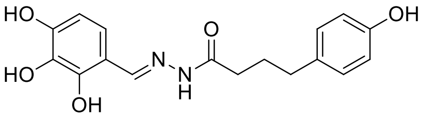

29e
(52.8%)

Figure S2. SAR-by-catalog study of compound 29 analogues. A total of five compounds were evaluated at a fixed concentration of 25 μM, with percent inhibition measured at the 90-minute endpoint. Percent inhibition is indicated in brackets.

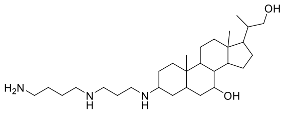

57a*
(78.3%)

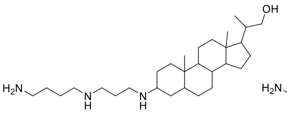

57b*
(99.6%)

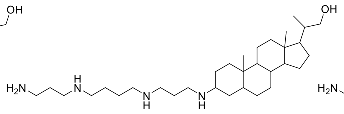

57c**
(99.7%)

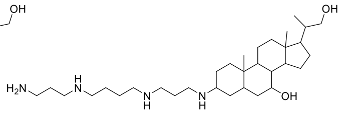

57d**
(98.2%)

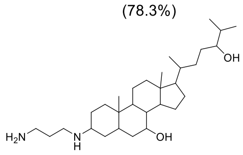

57e
(99.0%)

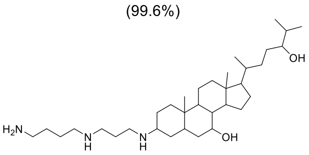

57f
(99.5%)

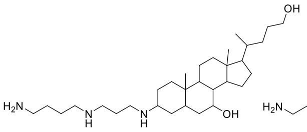

57g*
(97.9%)

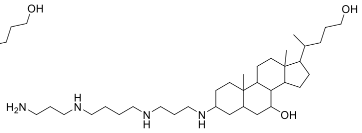

57h**
(97.8%)

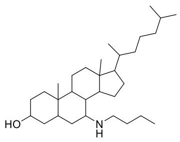

57i
(49.0%)

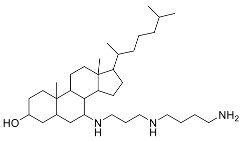

57j*
(99.9%)

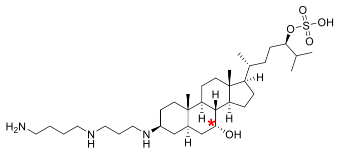

Squalamine

Figure S3. SAR-by-catalog Study of compound 57 analogues. A total of 10 compounds were evaluated at a fixed concentration of 25 μM, with percent inhibition measured at the 90-minute endpoint. Percent inhibition is indicated in brackets. *, ** indicates 3, 4 hydrochloric salt form, respectively. Squalamine, an analogue with well-defined stereochemistry, has also been investigated.

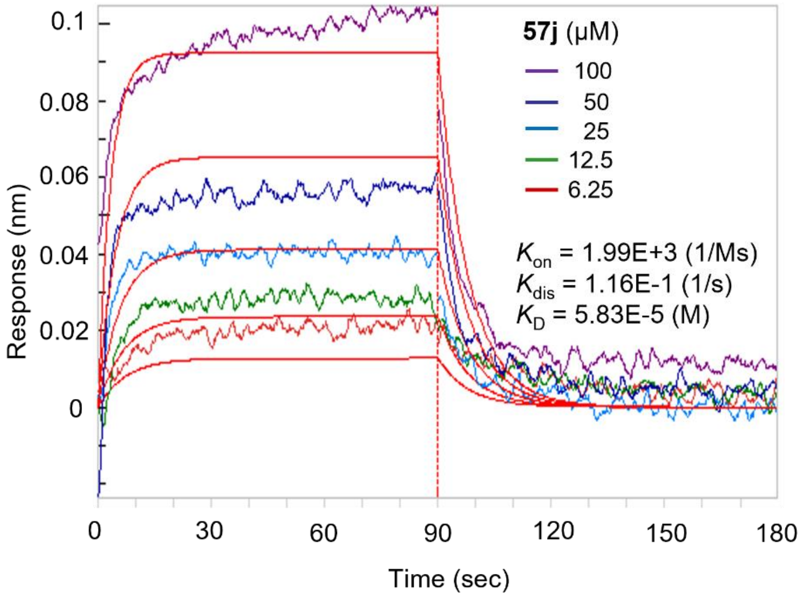

Figure S4. Binding kinetics of compound 57j by biolayer interferometry. Binding interactions were assessed using an anti-GST biosensor at five different concentrations of compound 57j.

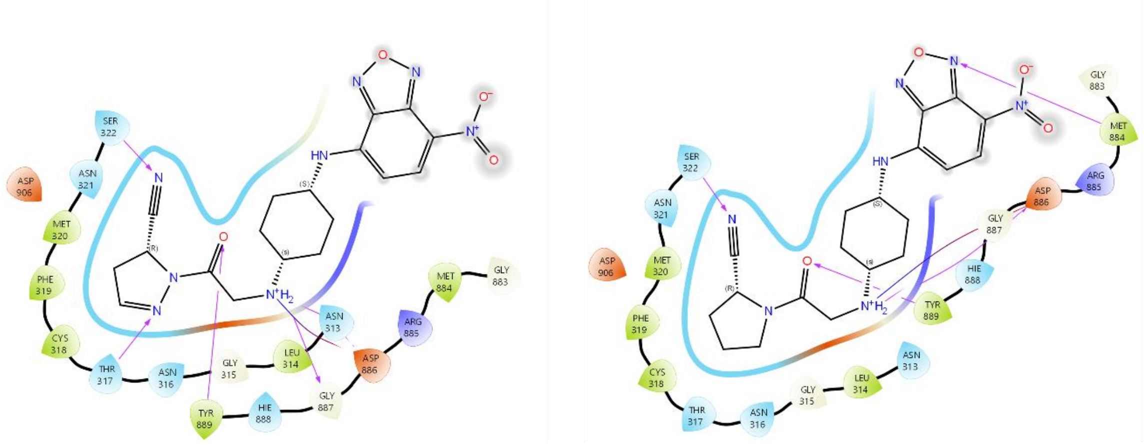

Figure S5. Protein-ligand interactions of 18g (left) and 18f (right). 18g is further stabilized with additional hydrogen bonding interaction with Thr317, which may explain the activity discrepancy observed.

Information Classification: General

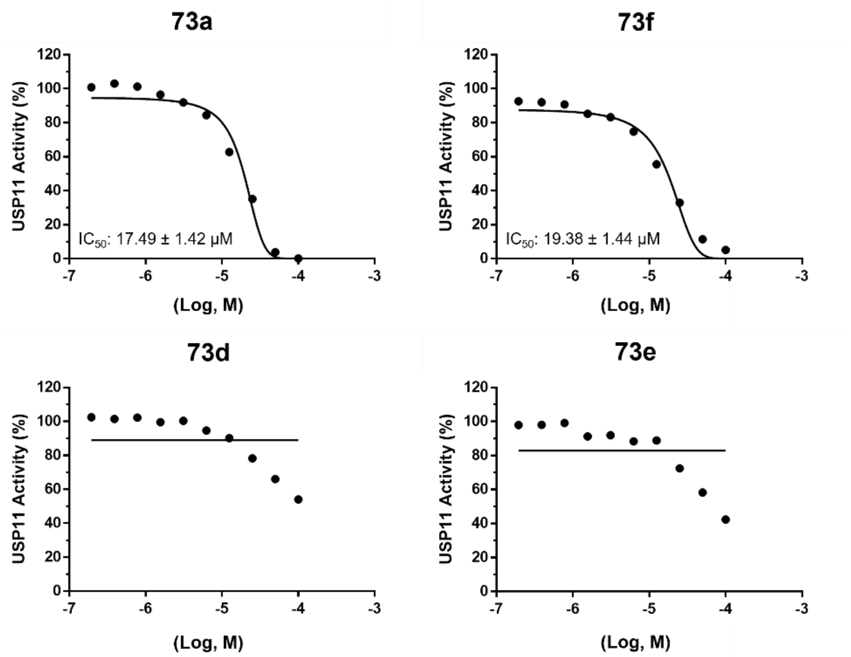

Figure S6. Dose-dependent inhibition curves of compound 73 analogues. Inhibitory activity of analogues 73a, 73d, 73e, and 73f against USP11 was evaluated using a fluorescence-based enzymatic assay. Percent inhibition was measured across a range of compound concentrations (Log[M]).

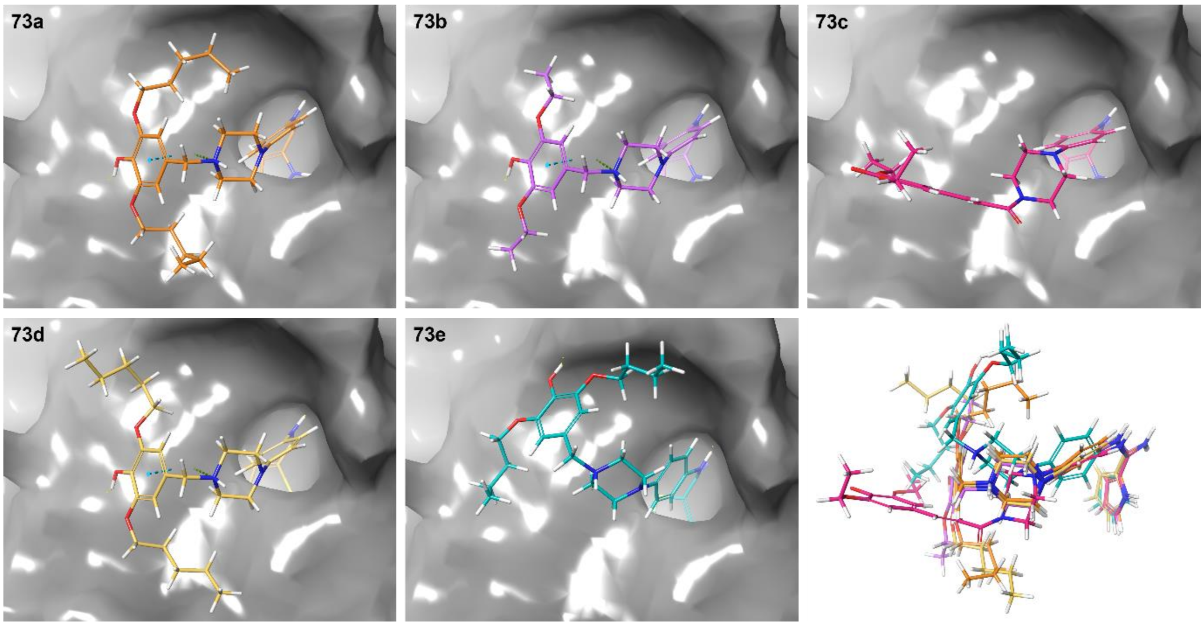

Figure S7. Binding poses of 73a to 73e predicted by molecular docking. The binding of the pyrrolo-phenylamidine moiety remains fairly constant where large deviations are observed for the 3,5-dialkoxy-4-hydroxyphenyl. Superposition of the ligands are depicted in bottom right.

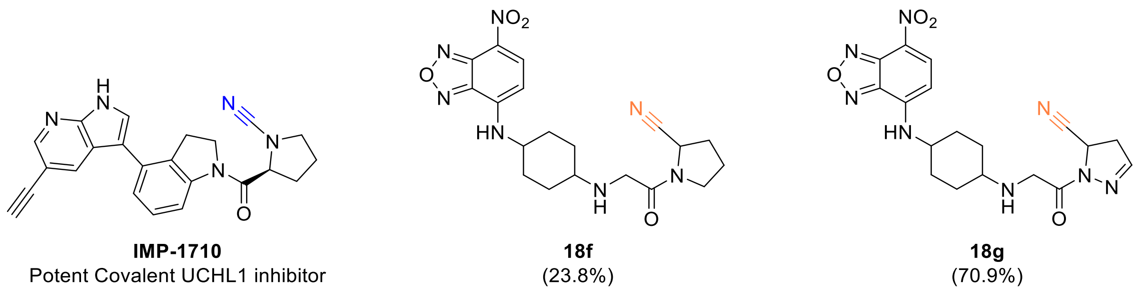

Figure S8. Structural similarity between IMP-1710 and 18f. The nitrile of IMP-1710 acts as an electrophilic warhead.

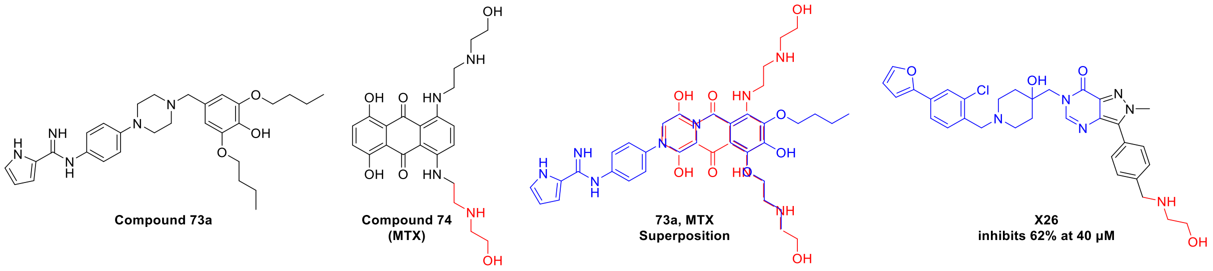

Figure S9. Potential structure features commonly found for USP11 inhibitory activity. X26 shares structural similarity with both compound **73a** and **74**, mitoxantrone.
